# Topical Nepafenac in Treatment of Acute Central Serous Chorioretinopathy

**Published:** 2013

**Authors:** Zeynep Alkin, Ozen Ayranci Osmanbasoglu, Abdullah Ozkaya, Gonul Karatas, Ahmet Taylan Yazici

**Affiliations:** Beyoglu Eye Training and Research Hospital, Bereketzade Cami Sok, Istanbul, Turkey

**Keywords:** Topical Nepafenac, Acute Central Serous Chorioretinopathy, Central Foveal Thickness, Visual Acuity, LogMAR, Subretinal Fluid

## Abstract

This study had been performed to investigate the anatomic and functional outcomes of nepafenac 0.1% therapy in acute central serous chorioretinopathy (CSC). The medical records of 30 patients with acute CSC were reviewed for a total of 31 eye charts. Seventeen eye records of 16 patients who were treated with topical nepafenac 0.1% three times daily for four weeks and continued until complete resolution of subretinal fluid were appraised. Fourteen patients with acute CSC (a total of 14 eye records) who did not receive treatment served as the control group also had been recorded. The proportion of eyes with complete resolution of subretinal fluid, serial changes in the mean best corrected visual acuity (BCVA), and the mean central foveal thickness (CFT) at 6 months of therapy were the outcomes measured. Mean age was 42.6±8.2 years in the treatment group and 41.1±7.1 years in the control group (*p*=0.85). At 6 months, 14 eyes (82.3%) in the treatment group and 6 eyes (42.8%) in the control group revealed a complete resolution in the subretinal fluid (*p*=0.02). In the treatment group, mean BCVA (LogMAR) significantly improved from 0.19±0.17 at baseline to 0.09±0.12 at 6 months (*p*=0.01). In the control group, mean BCVA (LogMAR) was 0.13±0.14 at baseline and decreased to 0.1±0.11 at 6 months (*p*=0.28). In the treatment group, mean CFT was 349±115 µm at baseline and significantly improved to 221±95 µm at 6 months (p<0.01). In the control group, mean CFT declined from 391±138 µm at baseline to 301±125 µm at 6 months (*p*=0.06). No treatment-related ocular or systemic side effects were observed. In conclusion, nepafenac 0.1% has the potential to treatment acute CSC. Further trials are warranted to study its safety and efficacy for this disease.

## INTRODUCTION

Central serous chorioretinopathy (CSC) is characterized by a serous detachment of the neurosensory retina at the posterior pole, which is caused by active retinal pigment epithelial (RPE) leakage (1,2). The disease has a favorable natural course with the spontaneous resolution of the neurosensorial detachment in association with improvement of visual function. However, it is very difficult to predict the prognosis of CSC, and in some cases, progressive visual loss may be seen (3,4). Thus, an intervention should be considered in CSC patients prior disruption of retinal layers. Gilbert et al. demonstrated that 51% of untreated patients experienced a single resolving attack, and 49% of them had a recurrent or chronic clinical course (5). Although the exact pathophysiology of CSC has not been clearly elucidated, the primary abnormality leading to RPE disruption and leakage is thought to be increased choroidal permeability (6). Studies using different imaging techniques have revealed the possible causes of abnormal permeability of the inner choroid. Ischemia and inflammation might lead to exudative changes within the choroid and the subsequent changes at the RPE (6-8).

 Nepafenac is a topical nonsteroidal anti-inflammatory prodrug that rapidly penetrates the cornea and is deaminated to form the active metabolite amfenac by intraocular hydrolases in the ocular tissues, including the ciliary body epithelium, retina, and choroid (9). Its bioactivation enhances intraocular penetration at the target sites and provides optimal distribution and longer duration in the cornea, iris, ciliary body, retina, and choroid (10). The effectiveness of nepafenac has been revealed in inflammatory diseases affecting the posterior segment of the eye such as uveitic and pseudophakic chronic cystoid macular edema (11).

 The purpose of this study was to evaluate the effect of nepafenac 0.1% in patients with acute CSC, and it was hypothesized that inflammatory process might be involved in the pathogenesis of the disease. 

## METHODS

The data were collected from medical records of consecutive patients who were admitted with CSC to the Retina Clinic of Beyoglu Eye Training and Research Hospital from December 2010 to July 2012. The treatment group comprised those with eyes treated by topical nepafenac 0.1%, and the control group comprised those without treatment. Approval for data collection and analysis was obtained from the ethics committee of the hospital. The study methodology was designed in accordance with the tenets of the Declaration of Helsinki. Written informed consent was obtained from the patients accordingly.All patients had compromised visual acuity caused by acute CSC diagnosed on fundus examination; fluorescein angiography (FA); indocyanine green angiography (ICGA) (Heidelberg Retinal Angiograph, Heidelberg Engineering, Heidelberg, Germany); and optical coherence tomography (OCT) (Stratus Tomographer, Model 3000; Carl Zeiss Ophthalmic Systems, Dublin, CA). All eyes had one or more focal areas of active angiographic leakage on FA and/or abnormal dilated choroidal vasculature and hyperpermeability on ICGA with neurosensory retinal detachment involving the fovea on OCT. Patients who received former treatment for CSC or had evidence of choroidal neovascularization or other maculopathy on examination and patients who had known or suspected hypersensitivity to any component of study medication or had any ocular pathology preventing nepafenac treatment were excluded. 

 A complete ophthalmic examination including best corrected visual acuity (BCVA) testing with standardized refraction using Early Treatment Diabetic Retinopathy Study (ETDRS) charts, applanation tonometry, and fundus examination with a 90 diopters indirect lens following mydriasis was performed at baseline and at 1, 3, and 6 months of therapy. 

 Drug therapy was started when there was no evidence of resolution in subretinal fluid after two consecutive monthly visits in the treatment group. All patients in this group received topical nepafenac 0.1% (Nevanac; Alcon Laboratories, Inc., Fort Worth, TX) 3 times in a day for at least 4 weeks and continued until subretinal fluid resolved. 

 The primary outcome measure was the proportion of eyes with complete resolution of subretinal fluid at 6 months of therapy. Secondary outcome measures were the serial changes in mean BCVA and mean central foveal thickness (CFT).

 Visual acuity was converted to a logarithm of the minimum angle of resolution (logMAR) for statistical analysis. Numerical variables were summarized using means and standard deviations while categorical variables were summarized using frequencies and percentages. The Mann-Whitney U test or chi-square test was used to compare variables between the two groups, and the Wilcoxon test was used to compare variables within the groups. The statistical evaluation was performed using SPSS (Version 16.0, SPSS Inc., Chicago, IL, USA). A *p* value of less than 0.05 was considered to be statistically significant.

## RESULTS

A total of 31 eyes from 30 patients with acute CSC were included in this retrospective, interventional, and controlled study. Seventeen eyes from 16 male patients with acute CSC were treated by topical 0.1% Nepafenac. The control group consisted of 14 eyes from 14 patients (11 male, 3 female) with acute CSC. The mean age was 42.6±8.2 years in the treatment group and 41.1±7.1 years in the control group (*p*=0.85). The demographic data and clinical findings of the two groups are shown in Table 1.

**Table 1:** Baseline demographics and clinical findings of the treatment group and control group.

	**Treatment group ** **(eyes n:17)**	**Control group ** **(eyes n:14)**	***p*** ** value**
Mean age ±SD^a^, years (range)	42.6±8.2 (26-59)	41.1±7.1 (29-52)	0.85
Gender, male/female (male %)	16/0 (100%)	11/3 (78.5%)	0.08
Mean baseline BCVA^b^±SD, logMAR^c^ (range)	0.19±0.15 (0.0-0.52)	0.13±0.14 (0.0-0.4)	0.29
Mean baseline CFT^d^±SD, microns (range)	349±115 (189-548)	391±138 (236-683)	0.08
Number of recurrences before therapy±SD (range)	1.5±0.7 (1-3)	1.2±0.4 (1-2)	0.46

a SD: Standard deviation;

b BCVA: Best corrected visual acuity;

c logMAR: Logarithm of the minimum angle of resolution;

d CFT: Central macular thickness

 At six months, complete resolution of macular subretinal fluid was observed in 14 of 17 eyes (82.3%) in the treatment group and 6 of 14 eyes (42.8%) in the control group (*p*=0.02). Three eyes in the treatment group and 8 eyes in the control group had no resolution of serous macular detachment, and these 11 eyes were assigned to have treatment with photodynamic therapy (PDT) or laser photocoagulation.

 In the treatment group, the mean baseline BCVA (LogMAR) changed from 0.19±0.17 to 0.16±0.15 at one month, 0.11±0.14 at 3 months, and 0.09±0.12 at 6 months (*p*=0.39, *p*=0.08, *p*=0.01, respectively). In the treatment group, there was a significant difference in mean BCVA at 6 months compared to the baseline. In the control group, the mean baseline BCVA (LogMAR) changed from 0.13±0.14 to 0.11±0.13 at 1 month, 0.11±0.12 at 3 months, and 0.1±0.11 at 6 months (*p*=0.43, *p*=0.39, *p*=0.28, respectively). In the control group, mean BCVA did not illustrate a significant difference throughout the follow-up period. No significant difference was observed between the two groups at baseline and at 1, 3, and 6 months (*p*=0.29, *p*=0.33, *p*=0.97, *p*=0.89, respectively). 

**Figure 1:**
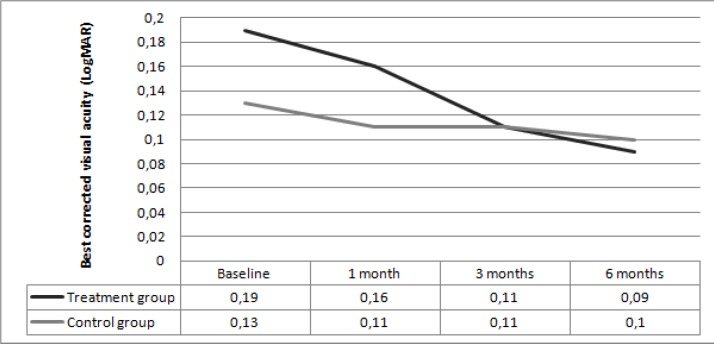
Change in best corrected visual acuity (logMAR) from baseline and to the 1-, 3-, and 6-month follow-up in the treatment group and control group.

**Figure 2:**
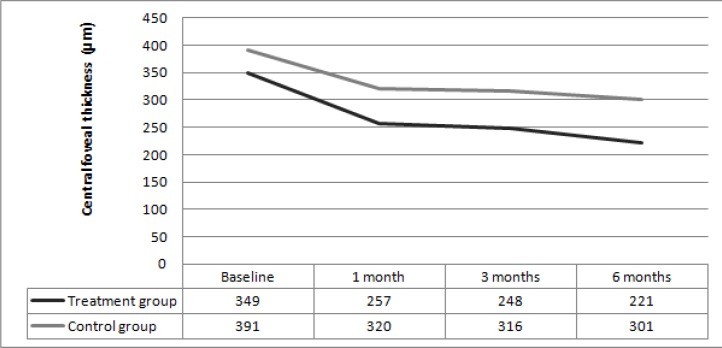
Change in central foveal thickness (µm) from baseline and to the 1-, 3-, and 6-month follow-up in the treatment group and control group.

 In the treatment group, the mean baseline CFT significantly decreased from 349±115 µm to 257±120 µm at 1 month, 248±101 µm at 3 months, and 221±95 µm at 6 months (*p*<0.01 for all comparisons). In the control group, the mean baseline CFT decreased from 391±138 µm to 320±132 µm at 1 month, 316±130 µm at 3 months, and 301±125 µm at 6 months. However, the mean baseline CFT did not change significantly compared to the baseline (*p*=0.08, *p*=0.07, *p*=0.06, respectively). No significant difference was observed between the two groups at baseline and at 1, 3, and 6 months (*p*=0.37, *p*=0.13, *p*=0.12, *p*=0.09, respectively). Furthermore, no ocular or systemic side effects were observed in the treatment group during the follow-up period.

## DISCUSSION

In the present study, the results demonstrated that topical nepafenac 0.1% therapy has promising results for the treatment of CSC. This disease generally affects young people who are of working age, and visual symptoms such as blurred vision, metamorphopsia, micropsia, dyschromatopsia, central scotoma, and hypermetropization might interfere with their daily activities considerably. The disease usually resolves spontaneously within three months after onset (12). Currently there is no standard therapy for acute CSC, even though there have been many treatment modalities studied including laser photocoagulation, PDT, and pharmacological agents (13-21). Heretofore, laser photocoagulation of the extrafoveal leaking points has been attempted to treat acute CSC. Although laser photocoagulation may shorten the time of resolution of serous detachment, it does not have any beneficial effect on the final VA. Additionally, photocoagulation may induce iatrogenic choroidal neovascularization and/or damage to the foveal photoreceptors (13). Lately, PDT with verteporfin has begun to be utilized for treatment of acute CSC. Although PDT may be beneficial for the treatment of acute CSC by decreasing the choroidalhyperpermeability, it also may trigger alterations on the structure of choroidal vasculature (14). The patients with acute CSC usually have relatively good baseline VA; therefore, it is imperative to keep in mind the potential adverse events caused by PDT. Chan *et al*. treated 63 patients with acute CSC with half-dose verteporfin PDT or placebo PDT in an attempt to demonstrate the safety of PDT (22). Subsequent to a follow-up time of 12 months, 94% of the eyes exhibited complete resolution of serous macular detachment in the half-dose PDT group versus only 57% of the eyes in the placebo group. The results of former studies revealed that half-dose verteporfin PDT is an effective treatment option for acute CSC. Despite the encouraging results of PDT with half-dose verteporfin, the cost of verteporfin may limit the use of PDT in treating acute CSC. 

 Treatment of acute CSC with intravitreal injections of anti-VEGF agents has variable outcomes (15,16). Lim et al. demonstrated that VEGF and IL-8 levels were not elevated in the aqueous humor and plasma of CSC patients compared with a healthy group (23). In light of these findings, further investigations are required with regard to the mechanism and results of intravitreal anti-VEGF treatment in acute CSC. 

 Pikkel *et al*. demonstrated limited recovery in CSC patients with acetazolamide (17). In addition, its use is limited because of its potential side effects. It has been proposed that corticosteroid antagonists could be used for treatment of acute CSC such as mifepristone and ketoconazole. However, trials with these drugs have proven unsuccessful (18,19). Metoprololand propranolol, another treatment strategy with adrenergic receptor inhibitors, should be used very cautiously because of its significant side effects and potential morbidity (20,21).

 Former studies have suggested that choroidal ischemia and/or inflammation caused by nitric oxide, prostaglandins, and free radicals might be involved in the pathogenesis of CSC (6-8). Consequently, medications such as antioxidants or anti-inflammatories might be effective in decreasing the choroidal leakage especially in the early stages of CSC. Ratanasukon *et al*. administrated either high-dose antioxidant tablet or placebo tablets for 3 months or until complete resolution of subretinal fluid (24). An additional treatment with laser or PDT was performed if any fluorescein leakage persisted following 3 months. They found no statistical difference in terms of VA and CFT between the groups at the end of the third month, but the patients treated with high-dose antioxidants revealed less fluorescein leakage. In the present study, the results demonstrated that topical nepafenac 0.1% therapy has promising results for the treatment of CSC. Treatment resulted in complete resorption of subretinal fluid in the macula in 82.3% of eyes in the treatment group versus 42.8% in the control group. Chan *et al*. demonstrated that complete resorption of subretinal fluid occurred in only 57% of eyes with acute CSC in the control group of one study at 6 months (25). We propose that our treatment regimen is superior to the natural resolution of the disease. In terms of its safety profile, the use of nepafenac 0.1% is promising because neither ocular toxicity nor systemic adverse effects were observed. 

 The limitations of this study were the small number of patients and the short follow-up duration. The actual role of nepafenac in treating acute CSC needs to be further investigated in randomized controlled clinical trials with a longer follow-up and with an adequate number of patients to determine the efficacy of the drug.

 In conclusion, the present study suggests that topical treatment with nepafenac 0.1% might be an efficient therapeutic option instead of conservative management for active working patients who suffer from impairment of visual function. We found that topical nepafenac 0.1% therapy was effective in treating acute CSC, leading to visual improvement and resorption of subretinal fluid in most of the patients. Since this study was conducted only for the acute stage of CSC after less than 3 months of onset, we do not suggest the use of this therapy in place of conventional methods for the complicated or chronic CSC.
